# Silicon Nanomembrane Filtration and Imaging for the Evaluation of Microplastic Entrainment along a Municipal Water Delivery Route

**DOI:** 10.3390/su122410655

**Published:** 2020-12-20

**Authors:** Gregory R. Madejski, S. Danial Ahmad, Jonathan Musgrave, Jonathan Flax, Joseph G. Madejski, David A. Rowley, Lisa A. DeLouise, Andrew J. Berger, Wayne H. Knox, James L. McGrath

**Affiliations:** 1 306 Goergen Hall, Biomedical Engineering, University of Rochester, Rochester, NY 14627, USA;; 2 508 Goergen Hall, The Institute of Optics, University of Rochester, Rochester, NY 14627, USA;; 3 Rochester Water Bureau, 7412 Rix Hill Rd, Hemlock, NY 14466, USA;; 4 Department of Dermatology, University of Rochester Medical Center, 601 Elmwood Ave, Rochester, NY 14642, USA; 5 405 Goergen Hall, The Institute of Optics, University of Rochester, Rochester, NY 14627, USA;

**Keywords:** silicon nanomembrane, microplastics, ultrafiltration, municipal water

## Abstract

To better understand the origin of microplastics in municipal drinking water, we evaluated 50 mL water samples from different stages of the City of Rochester’s drinking water production and transport route, from Hemlock Lake to the University of Rochester. We directly filtered samples using silicon nitride nanomembrane filters with precisely patterned slit-shaped pores, capturing many of the smallest particulates (<20 μm) that could be absorbed by the human body. We employed machine learning algorithms to quantify the shapes and quantity of debris at different stages of the water transport process, while automatically segregating out fibrous structures from particulate. Particulate concentrations ranged from 13 to 720 particles/mL at different stages of the water transport process and fibrous pollution ranged from 0.4 to 8.3 fibers/mL. A subset of the debris (0.2–8.6%) stained positively with Nile red dye which identifies them as hydrophobic polymers. Further spectroscopic analysis also indicated the presence of many non-plastic particulates, including rust, silicates, and calcium scale. While water leaving the Hemlock Lake facility is mostly devoid of debris, transport through many miles of piping results in the entrainment of a significant amount of debris, including plastics, although in-route reservoirs and end-stage filtration serve to reduce these concentrations.

## 1. Introduction

Plastic debris can be found nearly everywhere, even to the point where we can define a geological epoch by its presence [1]. As plastic weathers and degrades in the environment, there has been an intensive effort in the last decade to better understand the impacts of microplastic debris in a variety of settings, including along waterways [2–4], in the air [5], in wildlife [6], and in humans [7,8]. Plastics themselves are heterogenous materials, with a wide range of complex chemistries, many of which can trigger toxicological endpoints, such as oxidative stress and cytotoxicity [9]. The implications of this persistent debris for human health are not yet known, but there is evidence that plastic nanoparticles can cross the blood–brain barrier in fish, causing behavioral changes [10]. Additionally, polystyrene microspheres (5 and 20 μm in diameter) were found in the liver, kidney, and gut of mice after 28 days of exposure, and 0.1 mg/day of exposure was enough to induce metabolomic alterations [11]. One estimate suggests the average American consumes between 39,000–52,000 microplastic particles annually [12].

A recent review of microplastic assessments in fresh water sources documents a range of reported microplastic concentrations spanning ten orders of magnitude [13]. It is likely that both the variable measurement techniques and the true variation in environmental pollution levels help explain this remarkable range of answers in the literature and underscores the urgent need for standard definitions and practices. These challenges are confounded at the sub-millimeter size scale, where microparticulate debris has the potential to be absorbed by the human body [8]. In this range, it becomes more difficult to isolate microplastics in environmental samples efficiently, increasing time and cost, ultimately making an assessment of human exposure prohibitively difficult. Manual-inspections that pull out fibers or size plastic ‘nurdles’ are not scalable methods for quantifying the amount of small debris (<20 μm) in the environment, as many millions of microparticles could be produced from a single millimeter-sized fiber [14]. Methods for inspecting plastic debris rely heavily on the sequestration and planarization of the debris to make it compatible with different forms of metrology (e.g., fluorescence microscopy, Raman spectroscopy, Fourier-transform infrared spectroscopy (FTIR), energy-dispersive X-ray spectroscopy (EDS)). An ideal way to prepare samples for these techniques would be to utilize a direct filtration method to concentrate the particulate mass for imaging and characterization. Effective machine learning techniques can help categorize particles given clear imaging criteria [15]. Simply drying a drop of liquid and immobilizing particles on a surface can create samples for characterization on a smaller scale, but this method cannot interrogate larger liquid volumes for sparse numbers of particles efficiently.

Here, we demonstrate that ultrathin silicon nitride membranes [16,17] provide a robust way to rapidly filter municipal water samples and capture a size-specific range of debris for a variety of inspection techniques. Silicon nanomembrane technology has been used in a variety of biosensing applications since their introduction over a decade ago [18–24]. Nanomembrane filters are extraordinarily efficient at separations due to their thinness (<400 nm thick), providing separations at low pressures. Over the last decade, the silicon nanomembrane platform has transitioned into silicon nitride materials that are more robust while still maintaining the ability to fabricate a variety of pore shapes and sizes (50–30,000 nm). In the context of detecting microplastics, silicon nitride nanomembranes also provide a plastic-free background for direct measurements on the filter after filtration, are inert to harsh chemical treatments (4M KOH, 3:1 H_2_SO_4_:H_2_O_2_ Piranha Etch) used to dissolve biological contaminants, and create low-background signals to common spectroscopic and conventional imaging modalities. Thus silicon ‘nanomembranes’ offer significant value to the study of microplastic contamination, particularly in the smallest size range.

Our study introduces a novel silicon nanomembrane-based filtration and processing assay to track the existence of microplastic debris along the route from the City of Rochester’s water production facility at Hemlock Lake, NY to the University of Rochester’s Goergen Hall tap and drinking fountain. The ability to rapidly process water samples to visualize debris, including plastics, provides new context to earlier reports of microplastic contamination in municipal drinking water. We found that the water produced by the Hemlock Lake facility was relatively free of debris (10 particles/mL), however, some samples along the water transport route measured >1500 particles/mL (with microplastics accounting for as much as 9% of all the debris). Debris, including microplastics, was particularly concentrated at the end of long interrupted stretch of pipes including the entrance to the University’s Goergen Hall, suggesting they are entrained en route to the taps of homes and businesses.

## 2. Materials and Methods

### 2.1. Water Processing and Sample Collection

**Figure 1.**
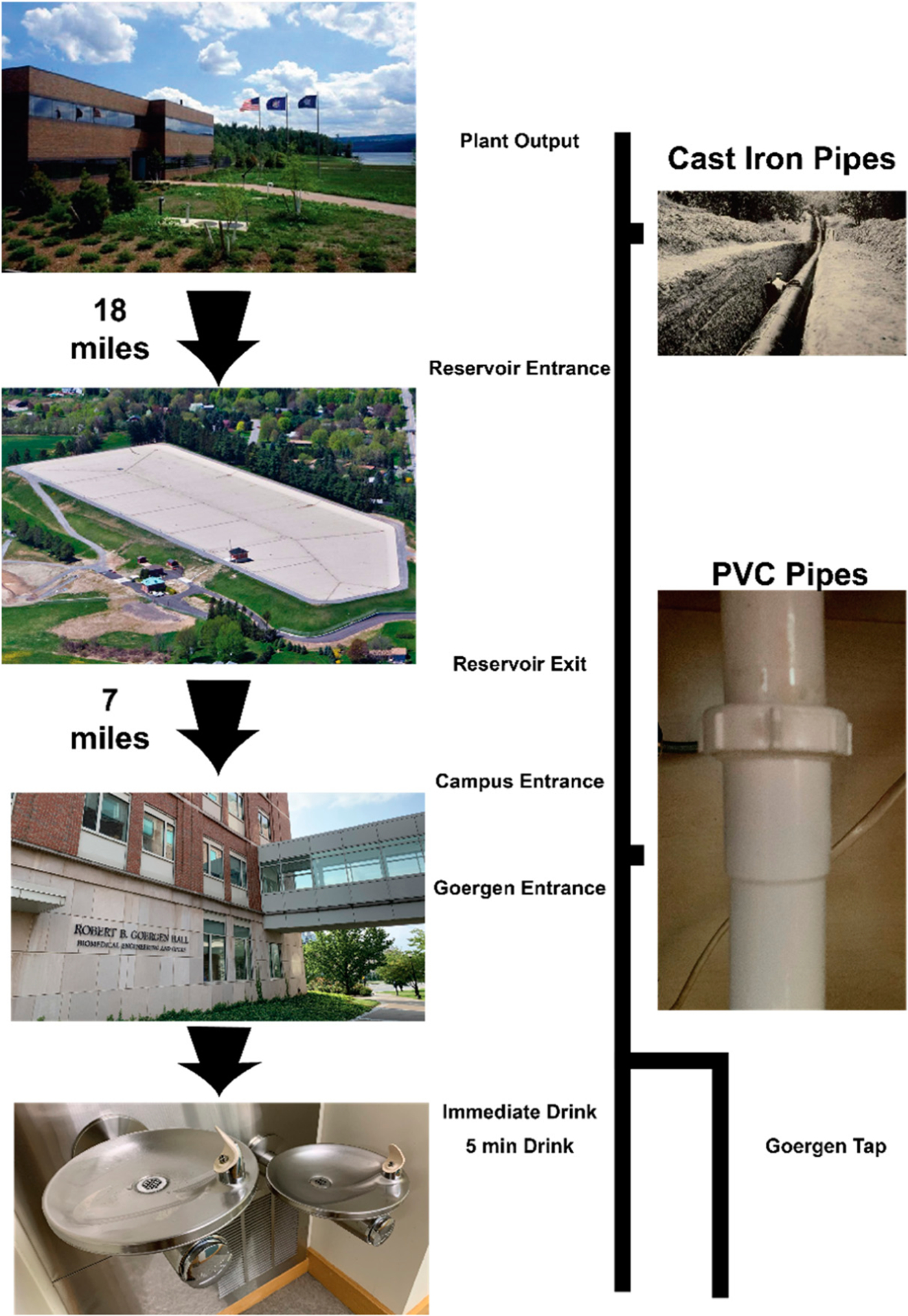
Sampling Path from Hemlock Lake to Goergen Hall. Water is transported through many different types of pipes and reservoirs before it is utilized at a drinking fountain on campus.

Samples were taken at various points along the water transport route from the Hemlock Lake water production facility to the Goergen Hall at the University of Rochester (Figure 1). Samples were collected on 2 July 2019 (between the Plant Output and Reservoir Exit samples) and 2 August 2019 (between the Campus Entrance and 5 min Drink samples). The Goergen Tap sample was collected on 16 December 2019. To ensure the sampling containers were not contaminating any of the sample collected, the outside and inside of containers were thoroughly rinsed with 100% ethanol stored in glass bottles, which we have previously found to contain no particulate. The containers and caps were sonicated for 1 h, then rinsed again with ethanol. After drying, 5 mL of ethanol was left in the container to confirm that the interior was airtight while being stored. As a control, we stored and processed 50 mL of ultrapure water (Invitrogen) in the same manner.

### 2.2. Sonication and Cleaning Protocol

All glassware, pipette tips, 1.5 mL conical Eppendorfs, and customized nanomembrane holder (SEPCON) components were sonicated for 15 min in 100% ethanol prior to use, then dried in a 70 °C oven. Glassware were covered with ethanol rinsed aluminum foil and all other items were stored in sterile, covered tissue culture petri dishes until use.

### 2.3. Graduated Cylinder Gravity Filtration

Lithographically patterned silicon nitride nanomembrane filters (5.4 × 5.4 mm silicon chip, three 0.7 × 3.0 mm rectangular windows, 8 μm slit widths 400 nm thick membrane, 6.3 mm^2^ active area) were produced by SiMPore Inc. (www.simpore.com, West Henrietta, NY, USA) [25]. These filters were then placed in SEPCON^™^ (SiMPore Inc., West Henrietta, NY, USA) units and sealed with silicone gaskets. The bottoms of 100 mL glass graduated cylinders were drilled out using a 3 mm diameter glass-drilling bit, then pressure-sensitive adhesive (3M) was used to attach a SEPCON device containing a silicon nanomembrane to the bottom of the cylinder, allowing gravity filtration to occur through both the cylinder and SEPCON device. Sample water (50 mL) was filtered through the silicon nitride nanomembranes, then the SEPCON units were removed and dried in a 70 °C oven. Membranes and debris were imaged under white light using a brightfield microscope (Nomarski DIC, 2.5625 pixels/μm), simultaneously in reflection and transmission mode at uniform lighting, or under white light using a dissection microscope (1.96 pixels/μm).

### 2.4. Dissolution and Washing Protocol

Eppendorf tubes (1.5 mL) were filled with 0.75 mL of 0.125 M Tris HCl. The SEPCONs containing filtered debris were then placed in the filled tubes. Next, 200 μL of 10% w/v Sodium Dodecyl Sulfate (SDS) and an additional 300 μL of 0.125 M Tris HCl were placed in the SEPCON basket. The Eppendorf-SEPCON unit was then heated to 95 °C and stabilized for 5 min at that temperature. An additional 200 μL of 14.6 M 2-mercaptoethanol was added to the SEPCON basket and the filters were left to process for 1 h inside of a fume hood. SEPCONs were removed and dried on a cleaned petri dish. Ultrapure water (Invitrogen) was heated to 90 °C. The heated water (750 μL) was used to load the SEPCON basket and left for five minutes. The SEPCONs were then gravity drained by lifting the SEPCON unit slightly out of the 1.5 mL tube, and this process repeated for 3 repetitions. After washing, the membranes were dried in a 70 °C oven, then imaged under white light using a brightfield microscope (Nomarski DIC, 2.5625 pixels/μm), simultaneously in reflection and transmission mode.

### 2.5. Nile Red Staining

Substrates were stained in situ with a lipophilic dye Nile Red (Abcam ab228553, 20 μL, 1 μg/mL), then imaged under an epifluorescent microscope (0.6107 pixels/μm). Stained debris was assumed to be plastic as described elsewhere [26].

### 2.6. Image Processing, Segmentation, and Quantification

Images were processed on a MacBook Pro (15-inch, 2019, 2.3 GHz 8-Core Intel Core i9, 16 GB Ram). The number of images analyzed for each replicate ranged between 9–36 images. Automatic segmentation of particulate from DIC imaging was achieved through the use of Trainable WEKA Segmentation [27], a plugin found in the Fiji [28] distribution of ImageJ [29]. A separate classifier was trained using 5–10 examples of 5 categories (defined as particle, slot, residue, membrane, and edge) identified on each sample. The classifiers were used to categorize images from each sample, then the probability map for each particle category was automatically generated (Minimum, Auto-Threshold, ImageJ) to create a binary map of debris. Fibers were extracted from the debris map, using an algorithm found in DiameterJ [30] (path 29), leaving only a sparse particulate map. The map was then processed with ImageJ’s watershed algorithm to separate aggregates. The particles were subsequently quantified using FiJi’s count particles plugin, capturing the individual particulate dimensions, which were then used to calculate a variety of physical information, such as particle volume with a simple model of an oblate spheroid.

Fluorescent images were cropped manually to the entire active area of the nanomembrane filter, then automatically thresholded (Minimum, Auto-Threshold, ImageJ) quantified using ImageJ’s watershedding algorithm to separate particles. Some outliers were manually removed from the data set (12/765 images) due to orders of magnitude changes in particulate concentration. Separately, fibers in images were counted by eye (2–3 independent volunteer counters) and particles of noticeable length were sorted into ‘large’ (>8 μm) or ‘small’ (≤8 μm) bins based on the relative size of the diameter of the fiber to the size of the slots in the silicon nanofilter (Supplementary Materials, Figure S1).

### 2.7. Elemental Analysis

We performed EDS (EDAX) on selected substrates using a Zeiss Auriga SEM. Substrates were sputter-coated with a 7 nm Au layer for improved backscatter imaging.

## 3. Results and Discussion

### 3.1. Water Transport Route and Filtration Apparatus

The City of Rochester’s drinking water is sourced from a Hemlock Lake, a minor Finger Lake located about 30 miles south of the city (Figure 1). The water treatment plant at Hemlock Lake withdraws water through a 30-foot-deep intake structure connected to a 60” diameter intake pipe that runs along the bottom of the lake. Water is passed through #4 mesh screens to prevent large objects and aquatic animals from entering the plant. Once the water enters the plant, it is treated with coagulants (aluminum chlorohydrate and cationic polymer), chlorine and CO2. Coagulated water passes through large chambers to allow flocculation (particle aggregation) to occur to enhance the filtration process. Filtration is achieved through a 6-foot column of anthracite and sand media. The output water stream (Plant Output) is then transported 18 miles through 19th century cast-iron and steel pipes, arriving at a reservoir in the town of Rush, NY. Samples were collected from water entering the reservoir (Reservoir Entrance) and exiting the reservoir (Reservoir Exit). The water is distributed throughout the city, including the University of Rochester. The Campus Entrance sample was collected from the pipes leading up to Goergen Hall, located underground between Park Lot and Goergen Hall. The Goergen Entrance sample was collected from a loading-dock access valve inside Goergen Hall itself, marking the last stop before the drinking fountain on the third floor. Internally, the final two samples for human consumption represents a use-case for drinking water at the fountain, immediately drinking it (Immediate Drink), compared with flushing the system (5 min Drink) and then collecting water. Because of filtration at the drinking fountain, a laboratory sink was measured (Goergen Tap) for comparison, as well as laboratory-grade ultrapure water (Control).

**Figure 2.**
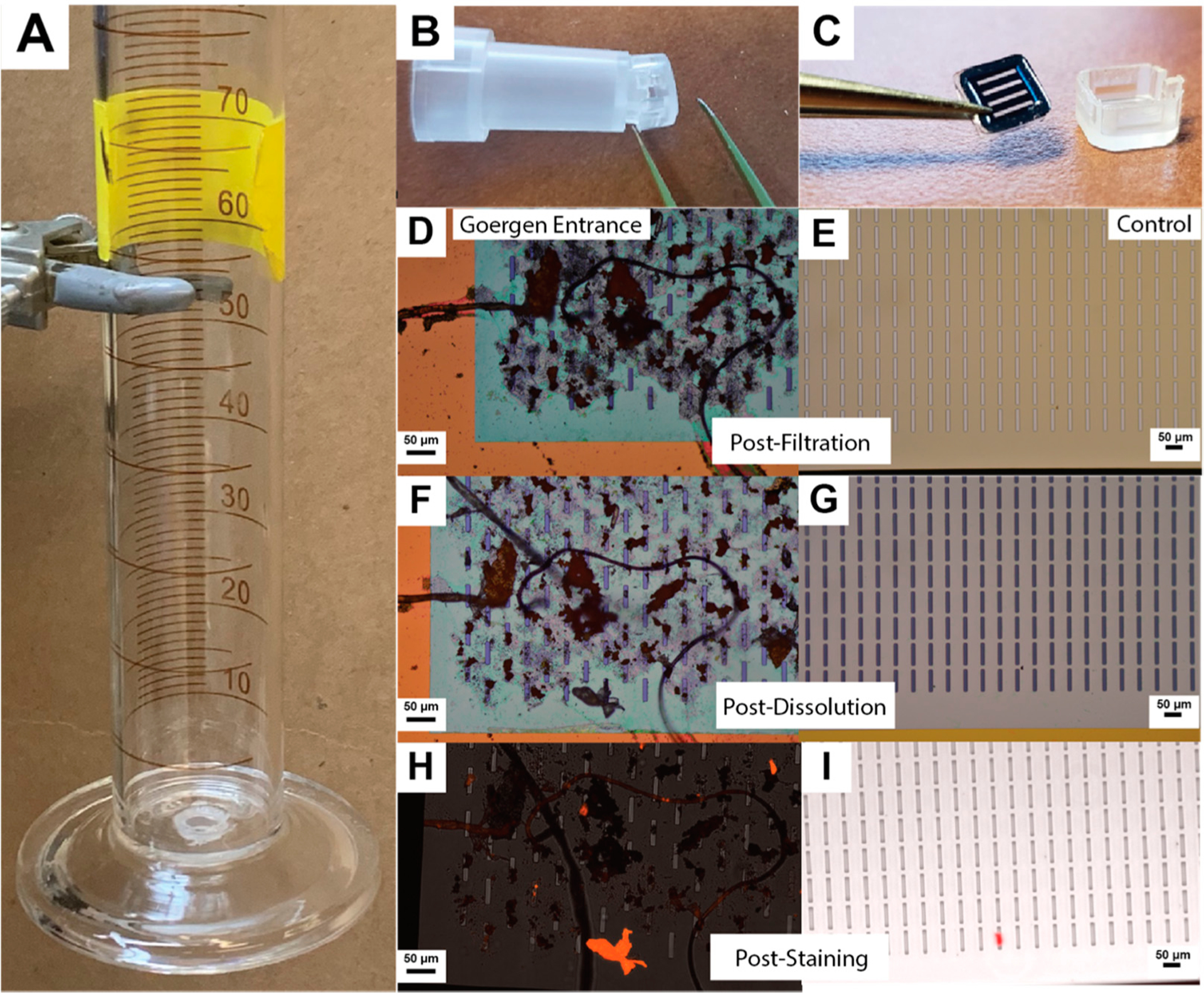
Direct isolation, dissolution, and staining of microparticulate debris. (**A**) Liquid samples (50 mL) are poured into 100 mL graduated cylinder apparatus and filtered through a silicon nanomembrane (5.4 × 5.4 mm chip, three 0.7 × 3.0 mm rectangular windows, 8 μm slits), attached at the bottom of the cylinder with a SEPCON adapter. (**B**,**C**) After filtration, the nanomembrane is removed from its protective housing and dried, (**D**,**E**) then imaged using a DIC microscope (transmission and reflection mode, white light). (**F**,**G**) After a dissolution protocol, the membranes are imaged again. (**H**,**I**) Membranes are stained with Nile Red dye as an indicator of plastic material and imaged using fluorescence (red false color, z-stack max projection).

We developed an apparatus that allows us to drain samples directly through a silicon nanomembrane filter for convenient image inspection of microparticulates (Figure 2), demonstrating the utility of capturing microparticulate using a silicon nanomembrane filter (SiMPore, Inc, Henrietta, NY). Due to their nanometer-scale thickness (and high porosity (>10%), silicon nanomembranes have very high permeability [20,31,32]. The membrane active areas are small (0.1–25 mm^2^) compared to polymeric or cellulosic membrane filters (Filter paper area = ~1500 cm^2^). Here, we use a 400 nm thick SiN film, lithographically patterned with 8 μm wide microslits (active area = 6.3 mm^2^) and simple gravity filtration to concentrate and capture debris on the surface. The starting head pressure of the liquid column in the graduated cylinder is 0.2 PSI (1.38 kPa), and filtrations of 50 mL drinking water sample volumes in this study typically take 10–15 min. As more debris is captured on the surface, flow diminishes. If the filtration cake occupies less than 60% of the membrane’s pores, the pressure difference is negligible (Supplementary Materials, Figure S2). After filtration, the membranes are extracted and imaged initially using white-light Nomarksi-DIC microscopy (8–10×). Then, the membranes are cleaned with an SDS-based mixture to gently remove organics on the surface of the microparticulate and then imaged again. Finally, the membranes with particulate are stained in situ using Nile Red stain (20 μL, 1 μg/mL) and imaged on an epifluorescent microscope.

### 3.2. Image Processing and Particle Enumeration

**Figure 3.**
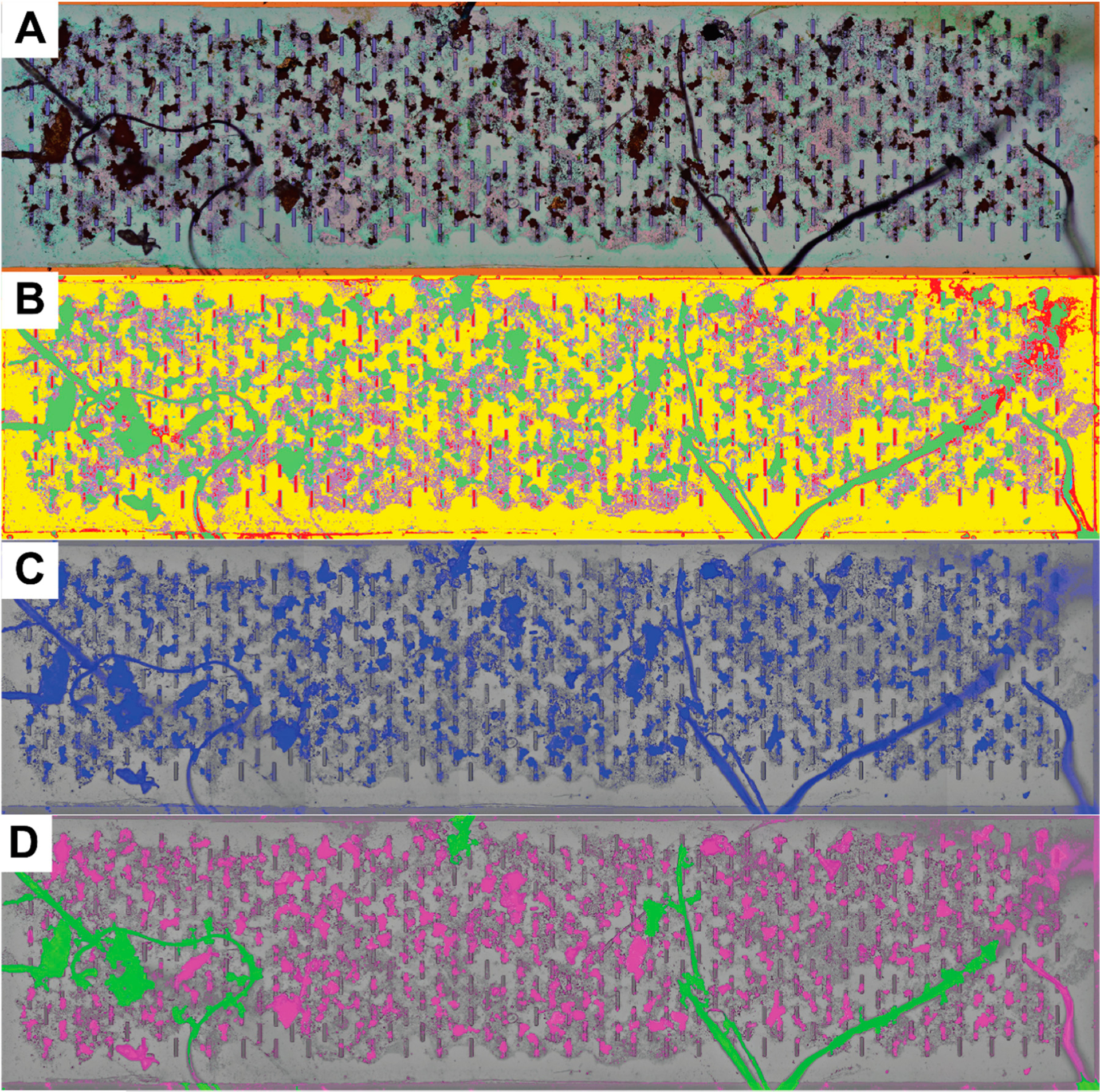
Semi-Automated image segmentation and particle counting. Individual images were merged in Photoshop to create the full membrane composite (Goergen Entrance). (**A**) Brightfield DIC images are used to train a classifier from 5–10 manually identified regions on each sample (Simple Random Forest [27], 100 iterations) (**B**) producing a set of probability maps for each classification (debris-green, slot-red, edge-cyan (thin features that are not visible here), membrane-yellow, and residue-purple). (**C**) The debris probability map is thresholded (Auto-threshold [33], Minimum 150 counts, overlaid in blue), then watershedded to separate aggregates. (**D**) Fibers (green) are extracted from particulate (magenta) analysis.

Figure 3 describes the image processing information for gathering the particulate information. Raw images (Nomarski-DIC/Phase) are categorized using an individual pixel classifier (simple random forest, 200 iterations, WEKA) trained from 5–10 manually identified regions on a single image from a sample. These classifiers are then applied to the full image set of the sample. WEKA segmentation is performed on scaled 2000 pixel-wide images, with classification times taking between 30 and 120 s/image with trained segmentation algorithms. Further analysis of a single image to perform particle/fiber segmentation and particle counting can take an additional 30 to 60 s per image. The classes chosen reflect characteristics of both the underlying silicon nanomembrane and the debris caught upon it. The background of the membrane (membrane—yellow) is fairly homogeneous, which makes it easier to also identify edges (edge—cyan), slits in the membrane (slot—red), and non-uniform residues (residue—purple). The debris (debris—green) class is then extracted and thresholded (blue) to identify fibers that span and reach beyond the individual image’s field of view (Diameter J). Each identified fiber (green) is counted and removed from processing the remaining debris, which are assigned as particulates (magenta). The particulates are then watershedded to separate aggregates, to better reflect their heterogeneous composition. The particles are then counted. In a similar manner, fluorescent images that were gathered post-staining with Nile red were auto-thresholded, to create the ‘debris’ classification, then analyzed using the same analysis path.

Individual pixel classifiers are useful for identifying regions of interest in an image, but additional guided image processing is necessary for discrimination of particles and fibers. Current limitations to the fiber processing algorithm necessitate that a fiber span the full field of view, as erode/dilation is used to find long-range fibrous structures; smaller fibers completely encapsulated in the field of view are counted as particulates (Supplementary Materials, Figure S3). Constraints on the aspect ratio of debris, commonly used as a fiber discrimination metric, would not work well for this filtration and analysis setup due to the potentially large number of particles (thousands) and their high overlapping potential, making watershedding operations a necessity (Supplementary Materials, Figures S3 and S4).

**Figure 4.**
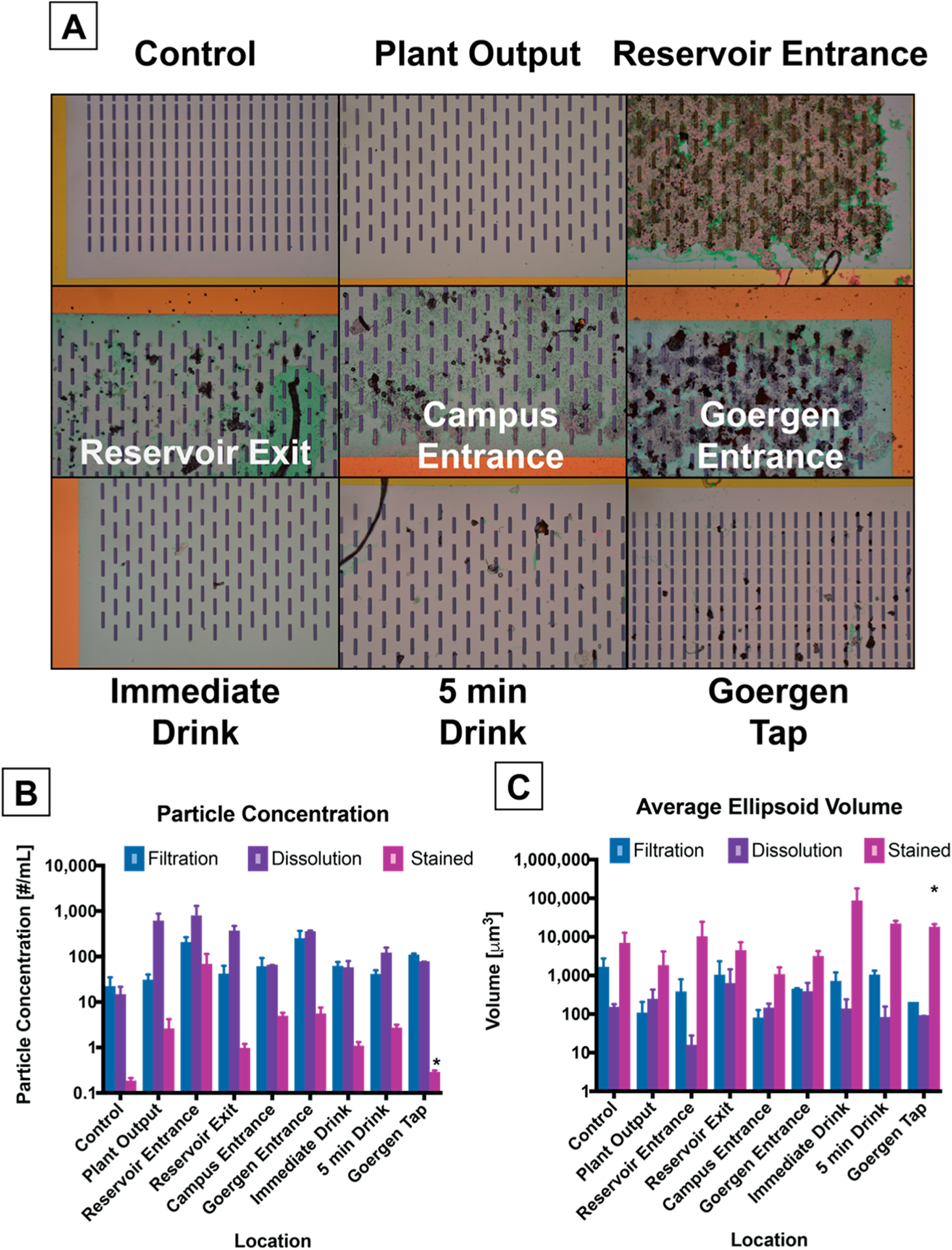
Particulate quantification along the water transport route. (**A**) Representative images of the captured particulate are shown (10× objective magnification, 8 μm wide slits). (**B**) Particle Concentration normalized to the volume of water filtered. (**C**) Average volume of a particle calculated from minor and major axis of image projection. *N* = 3 replicates, 9–36 images/replicate for dissolution and filtration stages, 1–2 whole field images for stained stage (*N* = 2 for asterisk [*]). Error bars are the standard error of the mean.

The full debris profile of this study is seen in Figure 4. Upon the exit of the water filtration plant, the water has little debris, but as the water is transported 18 miles through 150-year-old cast-iron pipes by gravity to a reservoir, the amount of particulate increases by roughly an order of magnitude. We also observe a reduction in particulate as the water exits the reservoir, potentially settling out over a residence time of 1.5–2 days. The water is transported another 7 miles to the University of Rochester where it is distributed to buildings on campus. A sharp increase is observed in the pipes entering Goergen Hall, and then decreases again at the output of the drinking fountain, which is filtered. The number of fibers caught is always much lower than the total amount of particulate (2–16 fibers/mL, automatically counted, Supplementary Materials, Figure S1). The dissolution step tends to increase the amount of counted particulate in addition to dissolving biological contamination (Supplementary Materials, Figure S5) while the staining process identifies a smaller subset of this debris, suggesting the smaller particles are typically fragments of organic debris or carbon from the environment, possibly previously coating the surface of the microplastic or larger organic particles. The largest significant difference in the amount of identified microplastic occurs at the Reservoir Entrance, while the highest concentration of debris occurs at the Goergen Entrance (Supplementary Materials, Figure S6). These values are compared to a control volume of ultrapure water (50 mL) which has been stored, transported, filtered, treated, and stained using the same methods.

The sizing of the debris is heavily favored toward the smallest particles that we can observe (approximately 1 μm in size), and also because of additional watershedding operation in the image processing that separate larger aggregates (Supplementary Materials, Figure S3). The dissolution cleaning stage also has the impact of disintegrating loosely bound aggregates into finer particulate, though there is potential for additional smaller material to be lost through the slits of the nanofilter.

In order to estimate the mass of debris captured on the nanomembrane, we applied a simple volumetric model to the identified 2D particulate images. Modeling each particle projection as an ellipsoid $\left(R_{1}=R_{2}=\frac{\text{Minimum}\;\text{Axis}\;\text{Length}}{2},\;R_{3}=\frac{\text{Minimum}\;\text{Axis}\;\text{Length}}{2},\;\text{Volume}=\frac{4\pi R_{1}R_{2}R_{3}}{3}\right)$ produces an average volume of positively-stained debris captured on the membrane on the order of 50,000–700,000 μm^3^. At the drinking fountain, this would produce a total plastic mass estimate of debris captured on the membrane as 300–700 ng (assuming a range of densities between ρ = 0.8–1.8 g/cm^3^). Larger microparticles will dominate these calculations, while particles that effectively minimize their aerial image projection or larger solid particles that were fragmented in image processing due to watershedding will necessarily create an underestimation of their volume by this method.

While the macroscopic appearance of drinking water may not change visibly, our exposure to small microplastic particulate from tap water can vary over orders of magnitude depending on the source of the water. Based on consuming 500 mL of water a day, the drinking fountain would permit 496 microplastic particles/day and 3–7 μg of microplastic load upon immediate use. If unfiltered, the pipes directly below the drinking fountain indicate 5.0 particles/mL (2522 particles/day) and 0.5–1.2 μg of microplastic load. Simple filtration appears to be effective in reducing the debris load, as there is a ~50% reduction in the amount of particulate at the drinking fountain immediately compared to the building’s source, however, more of these particulates appear to be plastic. These concentrations are in agreement with the higher reported values in literature for treated tap water (~10^6^ particles/m^3^), though there is a wide range of evidence reported (average ~10^4^ particles/m^3^) [13]. Many of the observed particles in pipes before the drinking fountain are rust and sand from the environment in which the water resides. Ultimately, the exploration of specific debris within this size range (<20 μm) of microparticulate can be more rapidly addressed without the need for laborious, time-consuming manual sorting and serial analysis.

### 3.3. Material Analysis

**Figure 5.**
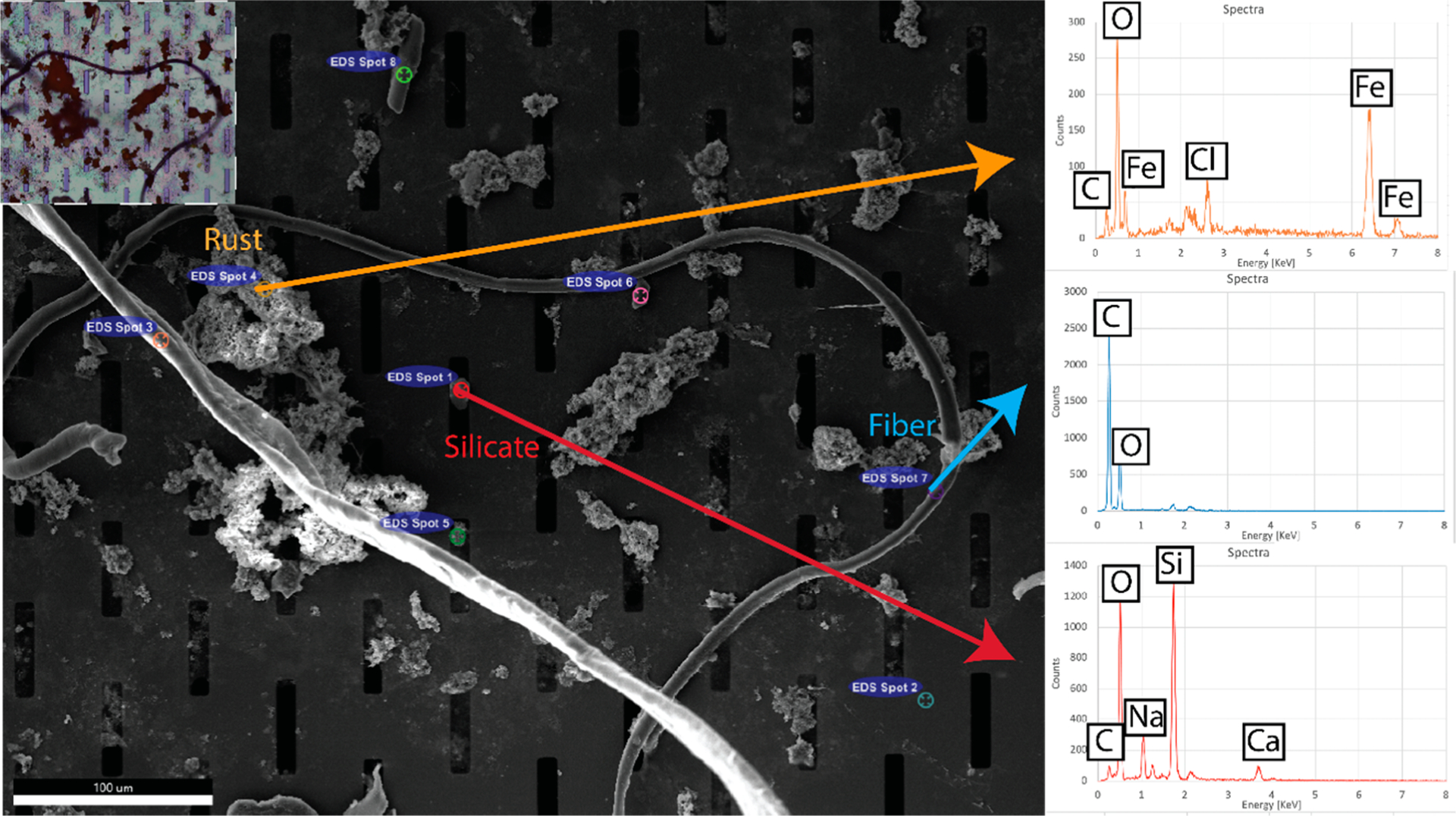
EDS measurement of debris captured on silicon nanomembrane. (**Inset**) Brightfield-DIC image of the region under inspection (Goergen Entrance). The placement of the microparticulate is well preserved, save an additional fiber laying down after transferring to the vacuum chamber of the SEM. Spectra taken from a number of individual microparticulates show a variety of elemental compositions, including likely identifications of rust and sand.

Although Nile red is an indicator of lipophilic materials, it does not tell us anything else about the material of the non-stained debris caught on the nanomembrane surface. To better understand the variety of debris caught on the surface, weused energy dispersive spectroscopy (EDS) to get an elemental composition of the microdebris. Figure 5 shows an example of debris caught underneath Goergen Hall (Goergen Entrance). There are a wide variety of elemental peaks observed; most commonly iron, carbon, calcium and chlorine. These spectra would commonly be found in rust, calcium scale, silicates, and other salts found on the surfaces of internal plumbing. Combined with hyperspectral imaging, or other material information from Raman spectroscopy (Supplementary Materials, Figure S7) and morphological imaging as seen here may create a more robust model of weathered particles.

**Figure 6.**
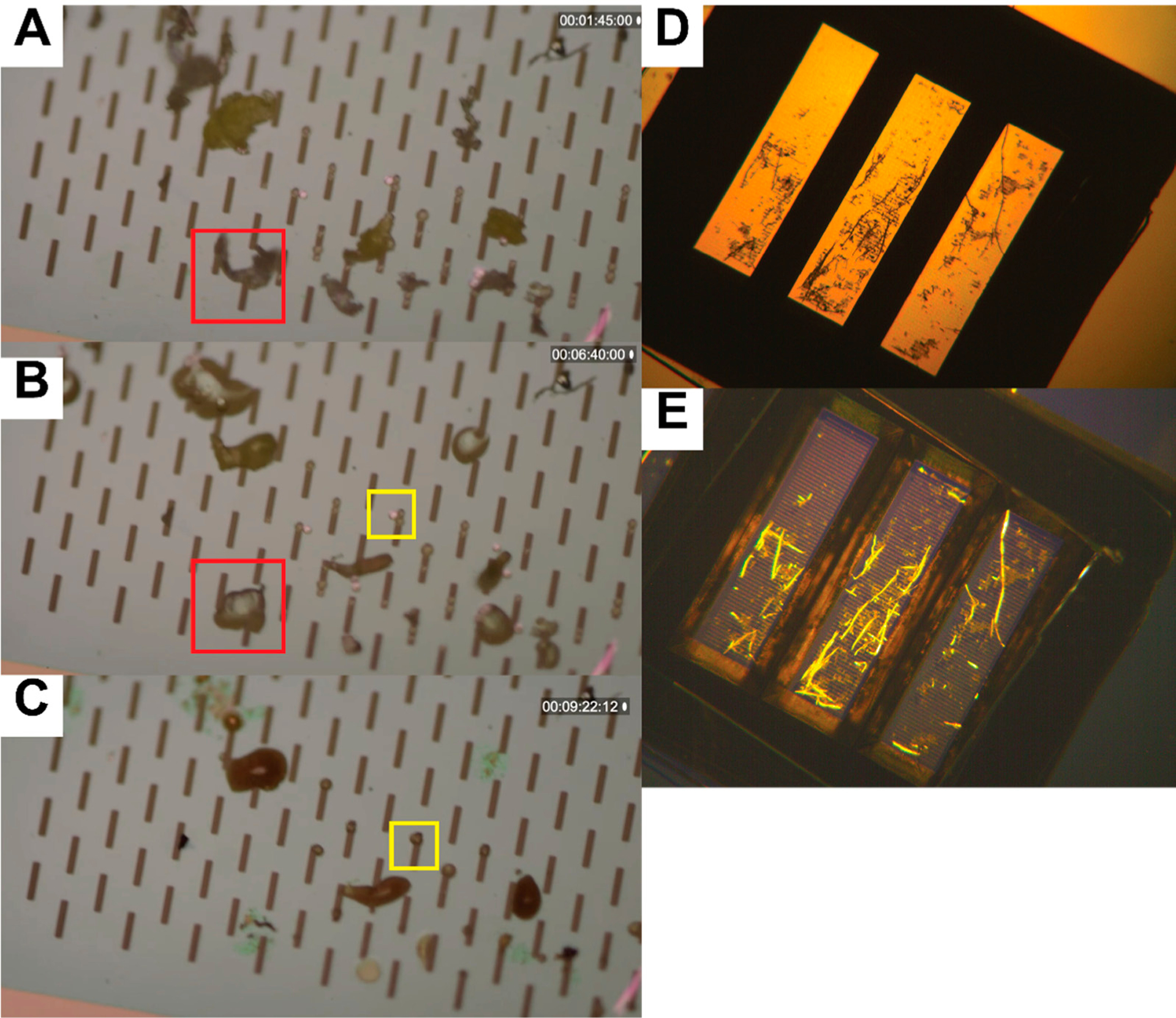
Alternative methods for material characterizations on silicon nanomembranes. (**A**–**C**) Microplastics differentiation by glass transition temperature. (**A**) Polystyrene beads and polyethylene shreds are captured on silicon nanomembrane (8 μm slit width). (**B**) The nanomembrane is heated on a ceramic resistor (350 °C surface), and the polyethylene shreds deform as the particles reach their glass transition temperature (red box). (**C**) After a few minutes, the polystyrene beads deform (yellow box) as these materials reach their glass transition temperature. (**D**,**E**) Microplastics differentiation by birefringence. A plastic tea bag was shredded and captured onto a silicon nanomembrane, then imaged under a bespoke polarizing microscope. (**D**) Viewed under yellow light, the plastic debris is not easily differentiable from tea leaf matter, however, (**E**) polarized illumination reveals birefringent properties of the tea bag shreds (yellow).

We also explored the possibility of evaluating material characteristics of plastic debris captured on the nanomembrane through their glass transition temperatures and intrinsic strains. While there are many different formulations of plastic materials, it is possible to discriminate functional material properties of the microdebris by applying heat treatments to the species in situ. Figure 6 demonstrates alternative methods to differentiate microdebris captured on the silicon nanomembrane. As the nanomembrane is not birefringent and is made of silicon-rich silicon nitride, it has the capacity to toleraterelativelyhightemperatures(meltingpoint1900°C)andremainundisturbed, whilemaintaining optical transparency. The ability to observe the debris as it is heated allows us to collect information about glass transition behavior for different materials. The phase transition of particles made from the same materials appears contemporaneously across the silicon nanomembrane (Supplementary Materials Video S1), indicating even heating across the microscopic field of view. This approach may help to differentiate between objects are similar in shape, size, and color without the expense of using a more sophisticated spectroscopic technique. Similarly, a common evaluation of plastics using polarimetry [34,35] can be used to distinguish stressed plastic fibers from otherwise dull matter surrounding it. Combining a multifactorial model, material library, and precise control of the temperature may eventually allow us to make material classifications based on this imaging platform alone.

## 4. Conclusions

We used silicon microslit filter technology to concentrate and purify microplastic debris from a variety of water samples taken along the water production and treatment route for our drinking water. This platform simplifies the sample preparation and quantification of small microparticulate through direct filtration, (5–60 min dependent on debris profile), staining (10 min), drying (10 min), imaging (5–15 min) and semi-automated machine learning for image analysis and particle classification (30–120 s/image). While the water leaves the producing plant relatively devoid of small debris particulate (sub 20 μm), transport through many miles of piping appears to entrain debris, while settling points and end filtration along this route reduce the amount of debris a person might consume. A majority of these particulates are not plastic although a significant fraction of them (up to 8.6%) were identified as microplastics using a lipophilic stain. This platform could be used in a variety of other contexts for environmental sampling of microdebris, such as a rapid quality control or a debris sensing mechanism. Furthermore, the usage of silicon nanomembranes enables many other sample preparation methodologies and complex metrologies that are unavailable to conventional membrane solutions. Lastly, the combination of small input volumes and membrane surface areas can allow for parallelization of sample processing and imaging in the future, leading to higher throughput analysis of small water volumes.

## Supplementary Material

Supplement

## Abbreviations

NPN: nanoporous silicon nitride
SiN: silicon-rich silicon nitride
pnc-Si: porous nanocrystalline silicon
EDS: energy dispersive X-ray spectroscopy
FTIR: fourier-transform infrared spectroscopy
SEPCON: separation container
DIC: differential interference contrast
WEKA: Waikato Environment for Knowledge Analysis
