# Supplementary material for: Silicon Nanomembrane Filtration and Imaging for the Evaluation of Microplastic Entrainment along a Municipal Water Delivery Route
Source: Sustainability. Author manuscript; Available in PMC 2023 Mar 17. (PMC10022737; doi:10.3390/su122410655)
Supplement: Supplement [file NIHMS1719770-supplement-Supplement.pdf]

sustainability

Supporting Information

Silicon Nanomembrane Filtration and Imaging for the Evaluation of Microplastic Entrainment along a Municipal Water Delivery Route

Gregory R. Madejski [1,*,†], S. Danial Ahmad [1,†], Jonathan Musgrave [2], Jonathan Flax [1], Joseph G. Madejski [1], David A. Rowley [3], Lisa A. DeLouise [1,4], Andrew J. Berger [5], Wayne H. Knox [2] and James L. McGrath [1,*]

1 306 Goergen Hall, Biomedical Engineering, University of Rochester, Rochester, NY 14627, USA; sahmad12@ur.rochester.edu (S.D.A.); Jonathan_Flax@urmc.rochester.edu (J.F.); jmadejsk@u.rochester.edu (J.G.M.); Lisa_DeLouise@urmc.rochester.edu (L.A.D.)
2 508 Goergen Hall, The Institute of Optics, University of Rochester, Rochester, NY 14627, USA; jmusgrav@u.rochester.edu (J.M.); wknox@UR.rochester.edu (W.H.K.)
3 Rochester Water Bureau, 7412 Rix Hill Rd, Hemlock, NY 14466, USA; David.Rowley@CityofRochester.gov
4 Department of Dermatology, University of Rochester Medical Center, 601 Elmwood Ave, Rochester, NY 14642, USA
5 405 Goergen Hall, The Institute of Optics, University of Rochester, Rochester, NY 14627, USA; andrew.berger@rochester.edu
* Correspondence: g.madejski@rochester.edu (G.R.M.); jmcgrath@bme.rochester.edu (J.L.M.); Tel.: +1-585-460-3113 (G.R.M.) +1-585-273-5489 (J.L.M.)
† These authors contributed equally to this work.

Automatic vs. Manually Counted Fiber Comparison

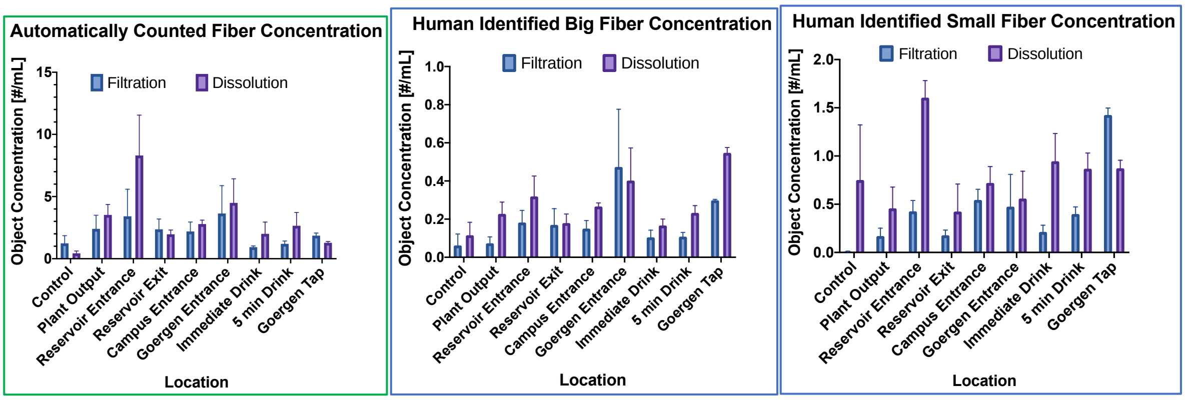

Figure S1 – Average number of automatically counted fibers vs human identified comparison. Error Bars are the standard error of the mean. Particles of noticeable length were sorted into 'large' (> 8 μm) or 'small' (≤ 8 μm) bins based on the relative size of the diameter of the fiber to the size of the slots in the silicon nanomembrane (8 μm). Overall, the automatically-determined fiber algorithm overreacts to larger particles on the boundaries of the image, leading to overestimations of the number density of fibrous structures.

Membrane Capacity

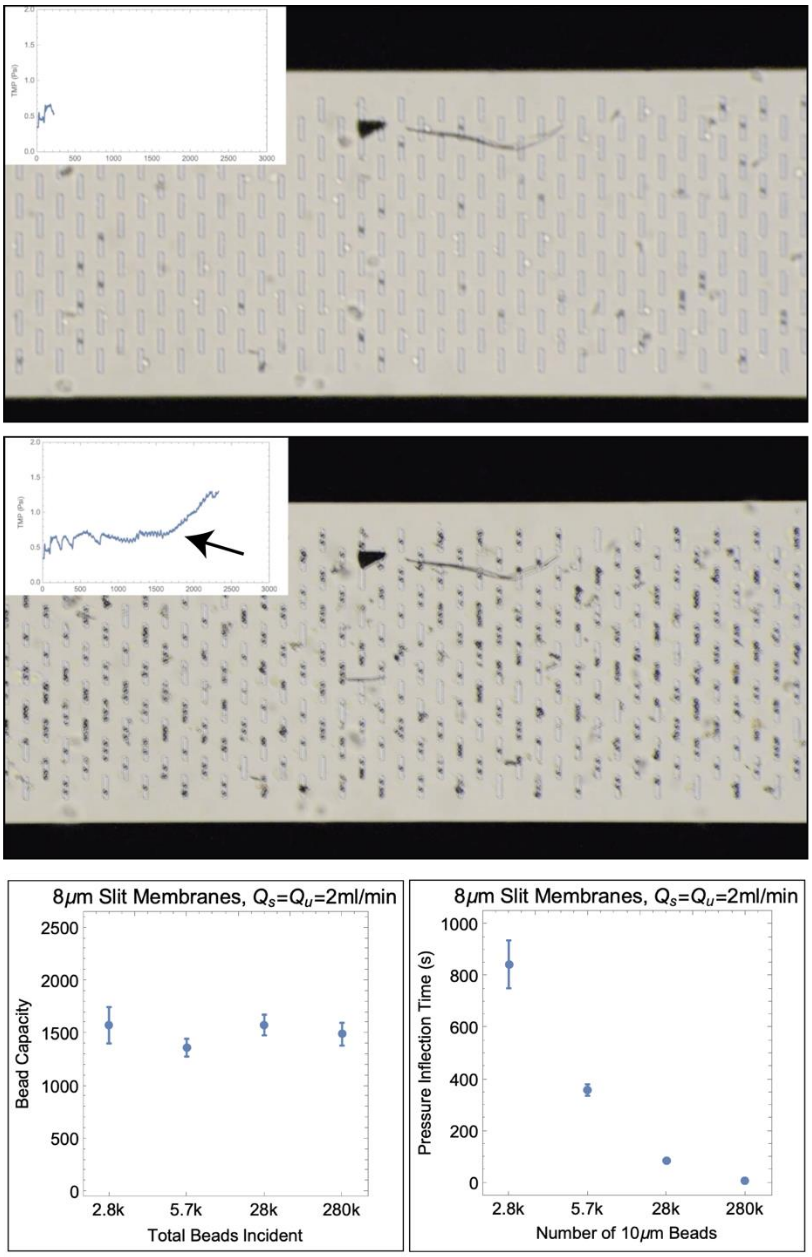

Figure S2 – Evolution of transmembrane pressure as membranes are fouled. A series of microslit nanomembranes with 8 μm slit widths were exposed to different doses of 10 μm polystyrene beads suspended in 50 mL of 1x PBS. Here we define occupancy as the number of beads present on the membrane surface normalized to the number of available positions in each slit and capacity as the percent occupancy at which a pressure inflection occurs. Transmembrane pressure was recorded across the nanomembrane as the liquid was filtered through the nanomembrane (2 mL/min), capturing beads and creating a rise in transmembrane pressure (black arrow). Transmembrane pressure is maintained as nanomembranes are fouled until a limit is reached. This is typically 60% fouling of the porous membrane area.

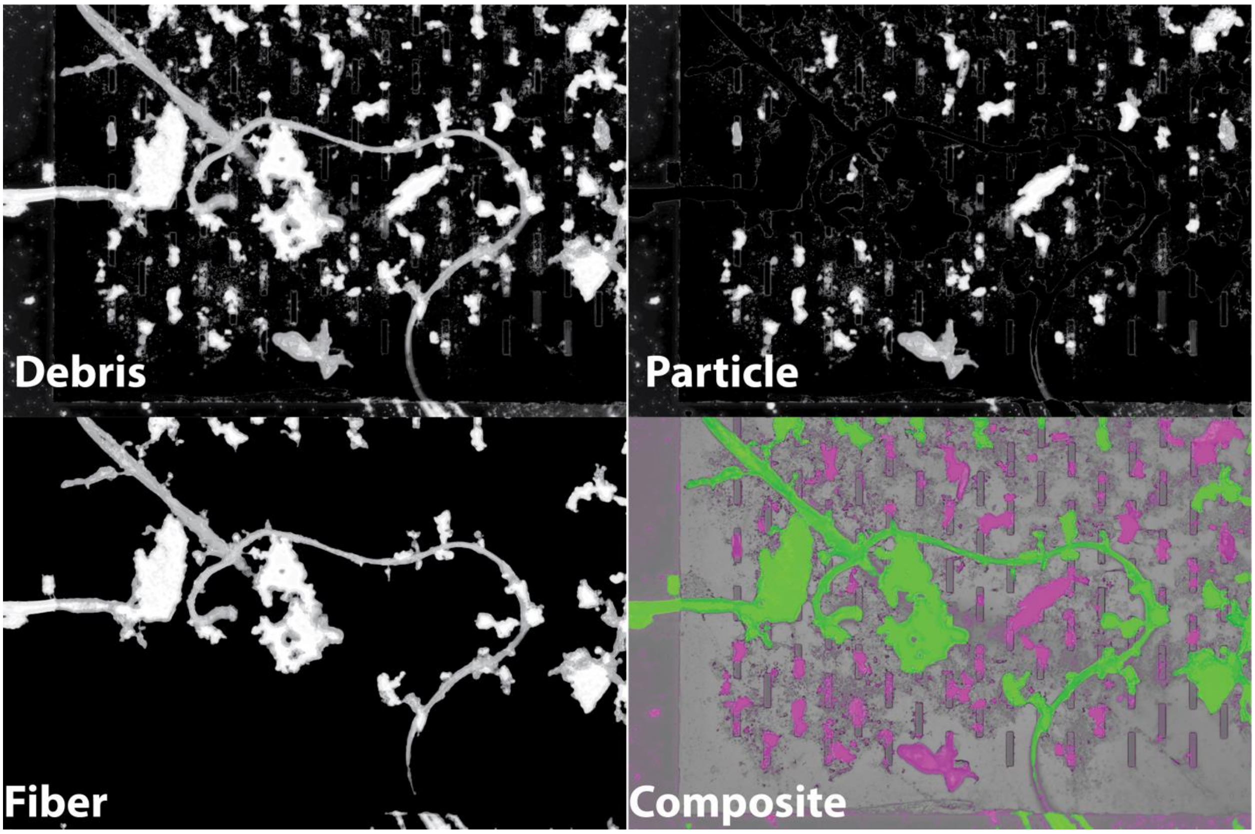

Figure S3 – Boundary Selection of Fibers. The Debris probability map is decomposed into particle and fiber segments by identifying long, contiguous image-spanning elements, starting from the border of the image. Debris contacting fibers are treated as part of the fiber itself. The Particle map subtracts the pixels of the Fiber Map from the Debris map, and a false color composite (green – Fiber, magenta – Particle overlaid with grayscaled DIC image) is shown.

Sizing of Aggregate Particles

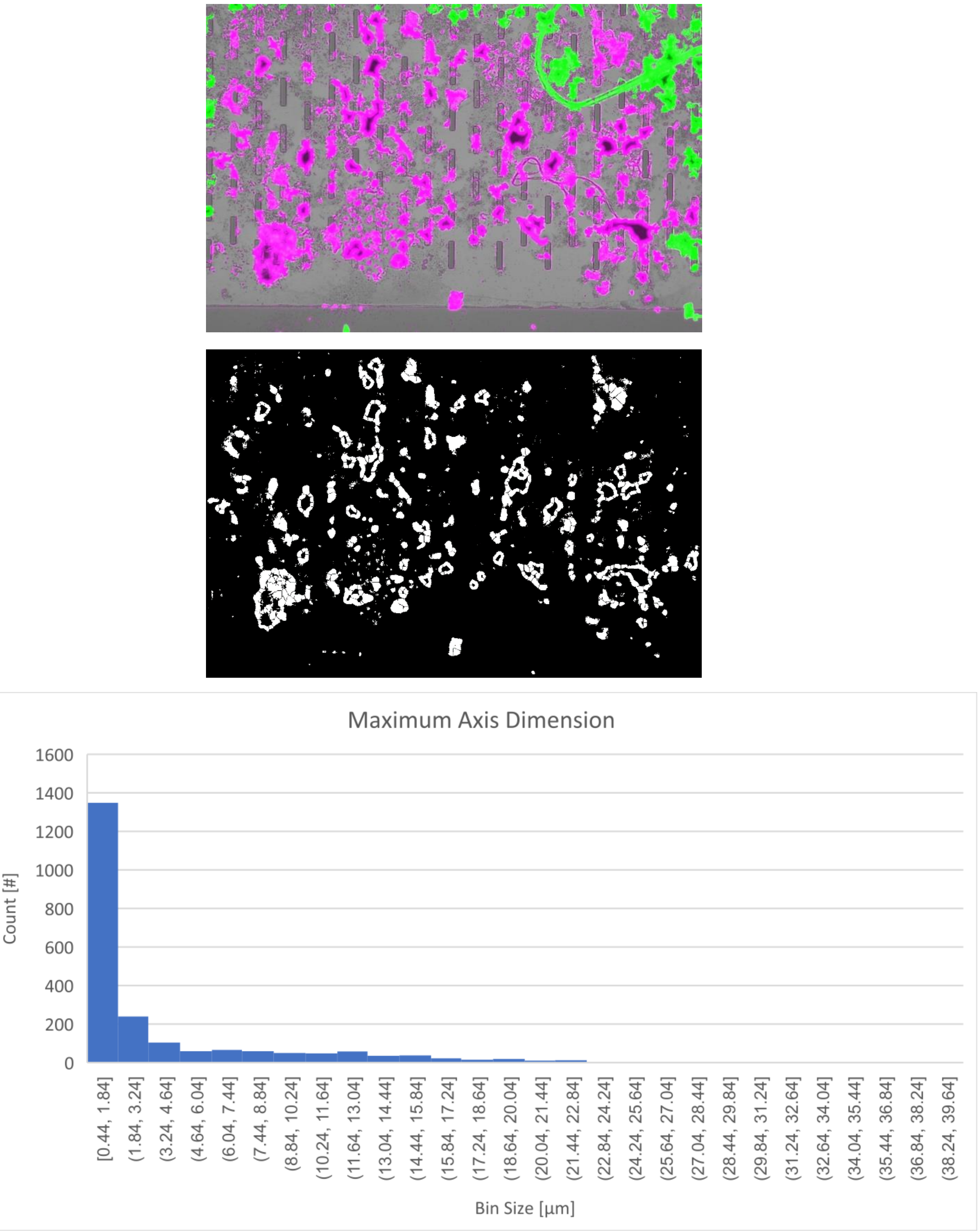

Figure S4 – Aggregate sizing of particulate. As the particulate probability map (magenta) is identified separately from the fiber probability map (green), the thresholded probability map of the classified particulate pixels (white) is watershedded, which separates larger aggregates into smaller particulate.

Dissolution Effectiveness

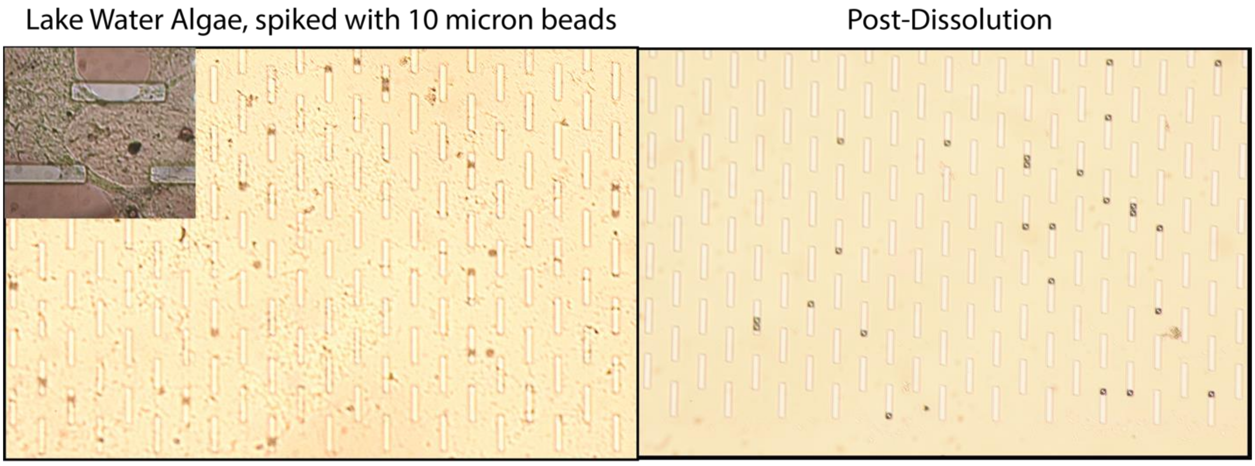

Figure S5 – Dissolution protocol removes organics with minimal disruption to plastics. 10 µm polystyrene spheres were introduced to 50 mL of water from Hemlock lake, then filtered through a silicon nanomembrane with 8 µm wide slits. After processing with the dissolution protocol, the spheres remain trapped on the nanomembrane. (inset) DIC image of algae on nanomembrane.

Significance Testing

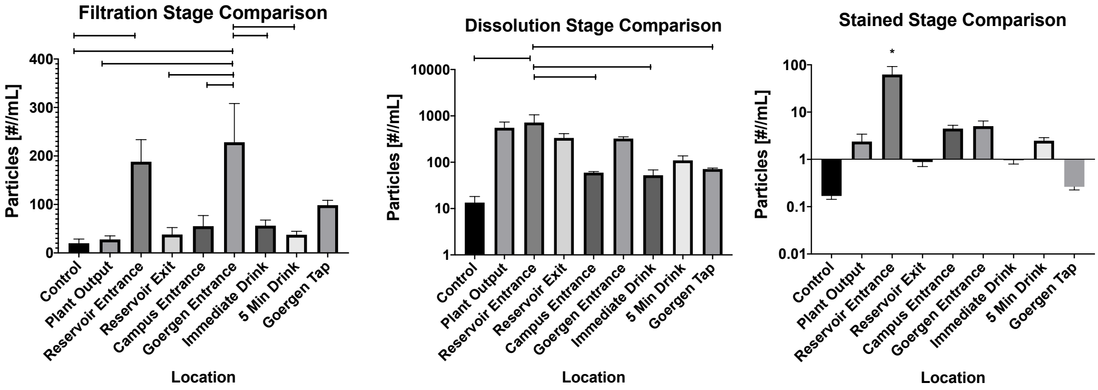

Figure S6 - Inter-location stage significance testing. (Graphpad 8, One-Way Anova, Tukey's post-hoc comparison, Dunnett T3 correction, alpha = 0.05). Lines indicate significance, the asterisk indicates that this location was significantly different compared to all other locations. The Goergen Tap sample is n=2, with error bars that are 50% of the value's range, while all other samples are n=3, and error bars are the standard error of the mean.

Raman Spectroscopy of Polystyrene Beads on Nanomembrane

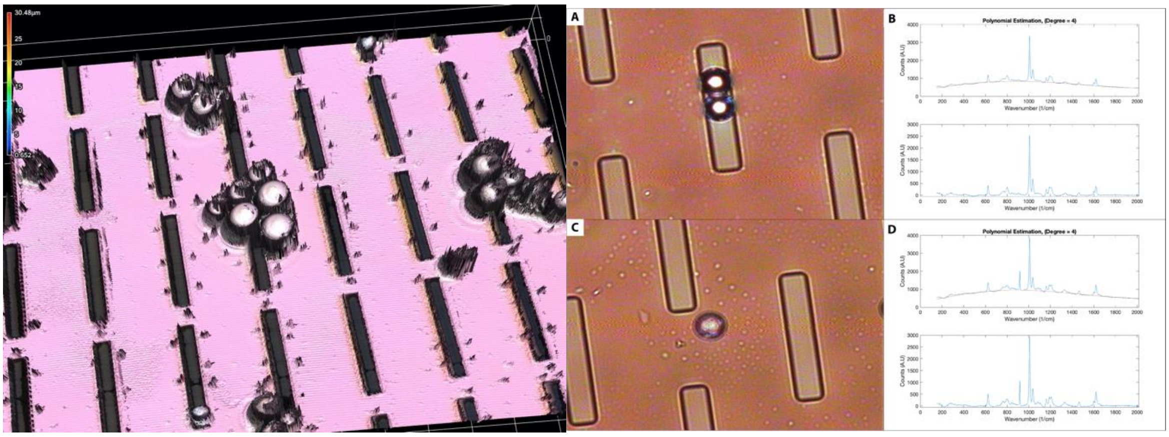

Figure S7 – Nominally 10 μm polystyrene spheres were captured on a silicon nanomembrane (400 nm thick, 8 μm wide slots) and imaged using a Keyence VK-X 3D laser scanning microscope. (A,B) Polystyrene Spheres (previously stained with Nile Red) caught in the nanomembrane slits have very similar Raman profiles (10s integration, background polynomial correction, degree = 4) to (C,D) those on the surface of the nanomembrane; the nanomembrane contribution to the Raman spectra is small.
